# Pre-anesthetic echocardiographic findings in children undergoing non-cardiac surgery at the University of Benin Teaching Hospital, Nigeria

**DOI:** 10.5830/CVJA-2016-006

**Published:** 2016

**Authors:** E Sadoh, Wilson, Ikhurionan Paul, Imarengiaye, Charles

**Affiliations:** Department of Child Health, University of Benin Teaching Hospital, Benin, Nigeria; Department of Child Health, University of Benin Teaching Hospital, Benin, Nigeria; Department of Anesthesiology, University of Benin Teaching Hospital, Benin, Nigeria

**Keywords:** pre-anaesthetic, echocardiography, children, noncardiac surgery, congenital rubella syndrome, cleft lip and palate, Nigeria

## Abstract

**Background:**

A pre-anaesthestic echocardiogram (echo) is requested for most non-cardiac surgeries to identify possible cardiac structural anomalies

**Objective:**

To describe the prevalence and spectrum of structural cardiac abnormalities seen in various non-cardiac conditions

**Methods:**

We carried out a retrospective review of pre-anaesthetic echos performed over five years on children scheduled for non-cardiac surgery. The requests were categorised according to referring specialities, and the biodata and echo findings were noted

**Results:**

A total of 181 children and 181 echocardiograms were studied, and 100 (55.2%) of the patients were male. Most of the children (87, 48.1%) with oro-facial clefts were referred from dentistry. Of the 181 children, 39 (21.5%) had cardiac abnormalities, most (34, 87.2%) of whom had congenital heart disease (CHD). Ophthalmic requests with suspected congenital rubella syndrome (CRS) had the highest prevalence of 8/12 (66.7%) while the lowest was oro-facial clefts at 15/87 (17.2%). Atrial septal defect was the commonest abnormality, found in 14 patients (35.9%)

**Conclusion:**

Pre-anaesthetic echo should be performed, especially for children with suspected CRS and other congenital anomalies, requiring non-cardiac surgery.

## Background

Patients requiring non-cardiac surgery, with suspected or symptomatic structural cardiac anomalies, may have increased peri-operative risk.^1^ This often necessitates a request for further cardiovascular evaluation, including echocardiography (echo) as part of the pre-anaesthetic evaluation to reduce anaesthetic risk.^1^

Other patients undergoing non-cardiac surgery may have conditions commonly associated with congenital heart diseases (CHD), such as children with cleft lip and palate,^2^ those with suspected congenital rubella syndrome (CRS) and other congenital malformations.^3^ The presence of congenital anomalies in one system may be associated with increased incidence of congenital anomalies in other systems.^4^ In particular, the presence of associated CHD increases the anaesthetic risk. It therefore becomes imperative that children with such suspected cardiac anomalies or those with conditions commonly associated with CHD and undergoing non-cardiac surgery are evaluated for the presence of cardiovascular anomaly.

From studies in Nigeria, the prevalence of CHD in children with cleft lip and palate ranges from 9.5 to 20%.^5^-^7^ The prevalence is higher in those with cleft palate than in those with cleft lip only. This relatively high prevalence of CHD in children with oro-facial cleft has prompted the policy in most centres of pre-anaesthetic echo for all such patients.

Congenital rubella syndrome is characterised by a triad of deafness, cataract and cardiac malformation.^8^ Affected children with cataract, which is the commonest ocular manifestation, would prompt presentation to the ophthalmologist. The children are referred for cardiovascular evaluation, including echocardiography, to confirm or exclude possible cardiac anomalies. Although a variety of CHDs have been found in children with CRS, patent ductus arteriosus (PDA) tends to be the predominant CHD reported.^9^

Adenotonsilar hypertrophy (AH) is a common cause of obstructive sleep apnoea syndrome and other sleep disorders in childhood.^10^ This is a common childhood presentation to ear, nose and throat (ENT) surgeons. Commonly associated with AH are ventricular hypertrophy and other rhythm abnormalities that may increase anaesthetic risk during adenotonsilectomy.^11^

Following the increasing availability of echocardiographic services in the country, some patients being prepared for non-cardiac surgeries are referred for pre-anaesthetic cardiovascular evaluation, including echo. The cost of echo is often high and this increases the cost of surgery. This study was conducted to describe the prevalence and spectrum of structural cardiac abnormalities seen in the children with various non-cardiac conditions, referred to our echo laboratory.

## Methods

The study was carried out in the echocardiography laboratory of a tertiary institution. It was a retrospective review of children aged from birth to 17 years who were referred either by the anaesthetist or surgeon for pre-anaesthetic echocardiographic evaluation. Some children were referred because they had congenital malformations for which they required surgery and therefore needed to exclude concomitant congenital heart defects. Others were referred for routine pre-anaesthetic cardiovascular examination, including echo, for conditions such as oro-facial clefts, congenital rubella syndrome and adenoidal hypertrophy. Occasionally, some were referred by the anaesthetist or clinician because of abnormal cardiovascular findings on clinical evaluation. The routine cases were often referred by surgeons while the ones with incidental findings were often referred by anaesthetists.

The children were grouped according to the referring departments/specialities, including restorative dentistry, ophthalmology, ENT and other units. The other units included paediatric surgery, and cardiothoracic and plastic surgical units. The haematology department referred one child with sickle cell anaemia for echo in preparation for stem cell transplantation. This case was added to the other units.

The echocardiography register/report sheet included information on age, gender and indication for the procedure, in addition to the echo findings. The study period was five years, between July 2009 and June 2014. Permission was obtained from the institutional ethics committee to use the patients’ data.

A transthoracic echo was performed by the paediatric cardiologist in the centre. Each child had two-dimensional, M-mode and Doppler examinations in multiple views. Left ventricular function was evaluated by measuring the fractional shortening (FS) and ejection fraction (EF) with the Teichholz method, using the Aloka Prosound SSD-4000SV (Aloka, Meerbusch, Germany). Analysis of the reports was done according to the recommendations of the American Society of Echocardiography.^12^ Any cardiac abnormality detected on echo was noted. This included CHD and acquired abnormalities such as ventricular hypertrophy and pericardial disease.

Right ventricular hypertrophy (RVH) was diagnosed when the free wall was > 5 mm, measured at end-diastole.^13^ Left ventricular hypertrophy (LVH) was diagnosed when the left ventricular posterior wall was > 13 mm, measured in systole.^14^ Other diagnoses were based on standard echo findings. The diagnosis of CRS was made using the World Health Organisation case definition.^15^ No confirmatory laboratory tests were done because the facilities were not available.

## Statistical analysis

The data were coded and entered into IBM-SPSS version 20.1 (Chicago, IL) and analysed using the same statistical tool. The frequencies of cardiac abnormalities are presented in simple percentages. Continuous variables such as age are presented as means and standard deviation (SD), or median and range if the range of values was wide. The median values of the ages, FS and EF between variables were compared using the Kruskal– Wallis test. The association between variables, such as cardiac abnormality and referring specialities, was compared using the χ^2^ test. Significance was set at p < 0.05 at 95% confidence level.

## Results

There were 181 children recruited over the study period, of whom 100 (55.2%) were males. The mean age was 3.0 ± 3.5 years with a range of two days to 16 years. The median age was 1.7 years.

The 181 children were referrals from dentistry (90, 49.7%), ENT (25, 13.8%), ophthalmology (19, 10.5%) and other units (46, 26.0%). The distribution of conditions requiring surgery for the referred children from the various departments is shown in Table 1.

**Table 1:** Conditions requiring surgery referred from the various departments

*Conditions*	*Number*	*Percentage*
Dentistry		
Cleft lip	38	21.0
Cleft lip/palate	29	16.0
Cleft palate	20	11.1
Facial cleft	2	1.1
Paroditis	1	0.6
Ophthalmology		
Cataract extraction	17	9.4
Strabismus	1	0.6
Ptosis	1	0.6
Eye agenesis	1	0.6
ENT		
Adenoidectomy	23	12.7
Meamatomy	1	0.6
Mastoidectomy	1	0.6
Other units		
Congenital limb abnormalities	10	5.5
Tracheo-oesophageal fistula	4	2.2
Anorectal abnormalities	3	1.7
Other congenital anomalies	11	6.1
Tumours	2	1.1
Stem cell transplantation	1	0.6
Other surgeries	15	8.3

ENT = ear nose and throat

The median ages of the cases according to the referring department/speciality are as follows: children referred from other units were 2.0 years (range: 2 weeks – 16 years), ENT 3.0 years (range: 1–13 years), ophthalmology 2.0 years (range: 3 months – 11 years) and dentistry 10 months (range: 2 days – 14 years). The difference between the median ages of patients referred by the various specialities was statistically significant (p = 0.01).

Of the 181 cases referred, 39 (21.5%) had cardiac abnormalities on echo. The abnormalities were CHD in 34 children (87.2%), and ventricular hypertrophy in five (12.8%). The 39 children with cardiac abnormalities consisted of 22 males (56.4%) and17 females (43.6%). The median age of the 39 children was 1.1 years (range: 2 days to 16 years). The distribution of the 39 children with cardiac abnormalities according to the referring departments is shown in Table 2. The highest percentage of cases with cardiac abnormalities was in patients referred from ophthalmology (9/20, 45.0%).

**Table 2:** Distribution of cases with cardiac abnormalities by referring department

**	*Number of*	*Number with*	**
*Referring speciality*	*cases referred*	*cardiac anomaly*	*Percentage*
Dentistry	90	15	16.7
ENT	25	5	20.0
Ophthalmology	20	9	45.0
Other units	46	10	21.7

ENT = ear nose and throat.

Of the 34 children with CHD, 31 (91.2%) had acyanotic CHD while three (8.8%) had cyanotic CHD. The commonest cardiac abnormality was isolated atrial septal defect (ASD) in 14 cases (35.9%), followed by isolated patent ductus arteriosus (PDA) in seven (17.9%). The cardiac abnormalities in the group with cyanotic CHD were tetralogy of Fallot, two cases (one was repaired), and one case of single ventricle. The distribution of cardiac abnormalities by referring department/speciality is given in Table 3.

**Table 3:** Distribution of the type of cardiac abnormalities by referring specialities

**	**	**	**	**	**	**	*Ventricu*	**	**
*Referring*	**	**	**	**	*ASD/*	*ASD/*	*lar hyper*	**	**
*speciality*	*ASD*	*PDA*	*VSD*	*AVSD*	*VSD*	*PDA*	*trophy*	*CCHD*	*Total*
Dentistry	7	3	3	–	–	1	–	1	15
ENT	1	–	1	–	–	–	3	–	5
Ophthalmology	3	4	1	1	–	–	–	–	9
Other units	3	–	–	2	1	–	2	2	10
Stem cell transplant									
Total	14	7	5	3	1	1	5	3	39

ASD = atrial septal defect, PDA = patent ductus arteriosus, VSD = ventricular septal defect, AVSD = atrioventricular septal defect, CCHD = cyanotic congenital heart disease, ENT = ear nose and throat.

Twelve children had suspected congenital rubella syndrome with cataract, and eight (66.7%) of these had cardiac abnormalities. They were all CHD cases and consisted of three with ASD (37.5%), three with PDA (37.5%), one with ventricular septal defect (VSD) (12.5%) and one with atrioventricular septal defect (AVSD) (12.5%).

Most (87, 96.7%) of the 90 children referred from dentistry had cleft lip or palate. Of the 87 cases, 38 (43.7%) had cleft lip only, cleft palate only was present in 20 (23.0%), and cleft lip and palate was present in 29 children (33.3%). Of the 90 cases, 15 children (16.7%) had cardiac anomalies, and all were CHD.

Of the three categories of oro-facial cleft, the highest proportion of CHD was found in children with cleft lip and palate (7/29, 24.1%), compared to children with cleft palate only (4/20, 20.0%), and those with cleft lip only (2/38, 5.3%). There was a significantly higher proportion of CHD in children with any form of cleft palate (12/49, 24.5%), compared to those with cleft lip only (2/38, 5.4%) (p = 0.019, OR = 5.8, 95% CI = 1.2–27.9). The distribution of type of CHD among children with different types of cleft lip and palate abnormalities is shown in Table 4.

**Table 4:** Distribution of the type of cardiac abnormalities in children with cleft lip/palate

*Type of CHD*	*Cleft lip*	*Cleft palate*	*Cleft lip/palate*	*Total*
ASD	1	2	3	6
PDA	–	–	2	2
VSD	–	2	1	3
PDA, ASD	–	–	1	1
TOF	1	–	–	1
Total (%)	2 (15.4)	4 (30.8)	7 (53.8)	13 (100.0)

CHD = congenital heart disease, ASD = atrial septal defect, PDA = patent ductus arteriosus, VSD = ventricular septal defect, AVSD = atrioventricular septal defect, TOF = tetralogy of Fallot.

No cardiac abnormality was seen in the case referred for echocardiography prior to stem cell transplantation. Among the children with ventricular hypertrophy, three with RVH were referred for evaluation for adenoidectomy. Two others with LVH were cases of Wilm’s tumour and Burkitt’s lymphoma, referred for evaluation for biopsy under general anaesthesia. The rest of the children from other units who had CHD consisted of two cases of omphalocoele, two with anorectal anomalies (Hirshsprung and imperforate anus), and a case of repaired tetralogy of Fallot requiring hernia repair.

Of the 46 children referred from other units, 28 (60.9%) had a form of congenital anomaly. Of the 28, five (17.9%) had CHD and none of the 18 without congenital anomaly had CHD. The difference was however not statistically significant (p = 0.14, OR = 7.1, CI = 0.37–137.20).

The median (range) FS and EF values of the study population were 38.0% (28.5–57.0) and 70.0% (56.8–81.1), respectively. Table 5 shows the FS and EF values of the study population by referring specialities. There was no statistically significant difference between FS and EF values by specialities (p = 0.48 and 0.70, respectively for FS and EF).

**Table 5:** Median values of fractional shortening and ejection fraction by referring specialities

*Referring*	*Fractional shortening*	**	*Ejection fraction*	**
*specialities*	*median (range)*	*p-value*	*median (range)*	*p-value*
Dentistry	38.0 (43.0–49.0)	0.48	70.0 (64.2–81.1)	0.70
ENT	36.5 (29.0–40.0)		67.6 (59.0–72.9)	
Ophthalmology	37.3 (34.0–40.0)		68.7 (65.4–72.0)	
Other units	37.5 (28.0– 47.2)		71.7 (56.8–79.8)	

*p-values for the difference in median fractional shortening and ejection fraction values between specialities. ENT = ear nose and throat.

The median (range) FS values of children with and without cardiac abnormalities were 35.0% (31.7–44.3) and 37.8% (28.0– 49.0), respectively (p = 0.64). The median (range) EF values of children with and without cardiac abnormalities were 67.3% (61.2–79.3) and 70.2% (56.8–81.1), respectively (p = 0.73).

## Discussion

In this study, 21.5% of children presenting for pre-anaesthetic echo for non-cardiac surgery had cardiac anomalies. The percentage in our study is lower than the 35% obtained in a study by Oyati et al. in Zaria,^15^ Nigeria, on children with non-cardiac congenital anomalies. The lower value in our study may have been due to the lower proportion of children with congenital anomalies in our study.

There is a higher risk of concurrent congenital anomalies, including CHD, in children with congenital anomalies.^4^ The high value of echocardiographically confirmed cardiac anomalies in our study supports the continued practice of echocardiography for such children, considering the increased anaesthetic risk that the presence of cardiac malformation may present.

The 16.7% prevalence of CHD in children with cleft lip and palate in our study is consistent with the 15% recorded by Otaigbe et al.^6^ in Port Harcourt, Nigeria, but lower than the 20% obtained in a similar study in Kano.^7^ The latter two studies consisted of small sample sizes and may have precluded drawing strong inferences from the studies, compared to our study with a sample size of 87 children.

The 16.7% prevalence of CHD we found is higher than the 9.5% seen in the study by Aimede et al.^5^ in Abeokuta, but much lower than the prevalence in the study by Sun et al.^16^ from eastern China. These various values may reflect the different influences of environmental factors on cleft lip/palate. Congenital cardiac anomaly was more prevalent in cases of cleft palate, and in a combination of cleft lip and palate, the prevalence was even higher. This is in keeping with previous works.^5^-^7^,^17^

Suspected CRS was the commonest ophthalmological referral for echo in this study and speaks to the endemicity of CRS in our environment. The high value of CRS in our study may be due to the lack of routine rubella vaccination in the nation. The highest prevalence (45.0%) of cardiac anomalies was found among children referred from ophthalmology. This may have been due to the high proportion of CRS among the referred children.

Cardiac malformations are particularly common among children with CRS, especially when the infection occurs early in pregnancy.^18^ In our study, 66.7% of children with suspected CRS had cardiac anomalies. This high value is consistent with the findings of 85.7 and 72% by Otaigbe et al.^6^ in Port Harcourt, Nigeria and Kyaw-Zin-Thant et al. in Myanmar, respectively.^19^

This finding also suggests that cardiac anomaly is more likely in children referred for cataract extraction than for any other non-cardiac surgical condition. This is because CRS is a common cause of childhood cataract. The number of suspected cases of CRS over a five-year period in this study (12) is higher than the seven cases seen by Otaigbe et al.^9^ in Port Harcourt, Nigeria. This may reflect the different levels of activity of the virus in different localities.

The cardiovascular anomalies in children with cleft lip/palate were all CHD. The spectrum of CHD consisted mostly of acyanotic CHD, as documented in the studies from Port Harcourt and Kano.^6^,^7^ The commonest CHD was atrial septal defect (ASD) followed by VSD. These CHDs were similarly reported in earlier studies from Nigeria and outside Nigeria.^6^,^7^,^17^ The preponderance of ASD may suggest that most of the children with cleft lip/palate would be asymptomatic and appear apparently normal on clinical evaluation. This further buttresses the need for echo evaluation prior to anaesthesia.

PDA and ASD were the commonest CHD in children referred from ophthalmological surgery, of whom most had suspected CRS. Most previous studies on children with CRS also demonstrated PDA and ASD as common CHDs.^8^,^9^ Most children referred from ENT were for adenoidectomy, which suggests that the condition is quite prevalent in children.

Only three of the 20 cases with adenoidal hypertrophy had ventricular hypertrophy. The low prevalence of ventricular hypertrophy in children with obstructive sleep airway syndrome has similarly been reported in an earlier study from Ibadan.^20^ It was recommended from the Ibadan study that cardiovascular evaluation be reserved for children with severe disorder. However considering the two cases (10%) of ASD among children referred from ENT in our study, it might be worthwhile continuing to request echocardiograms for all children with adenoidal hypertrophy, not only to identify ventricular hypertrophy but also to detect possible CHD in these children.

Most previous works studied the echo changes in children with cancers on chemotherapy.^21^,^22^ Ventricular echo indices appeared normal in cancer patients who were not on chemotherapy.^21^ In our study however, we noted the presence of left ventricular hypertrophy in the two oncology cases referred for echo. It is not clear whether the advanced stages of the disease were responsible for the findings in this study. It is important therefore to study this group of patients further to evaluate cardiac function, since the number of these subjects in our study was small. It is particularly important, as most patients present with advanced stages of cancer in our environment, and a number of them undergo anaesthesia for surgery to debulk masses or for open biopsy.

The children referred from dentistry were significantly younger than those from other specialities. This finding may speak to the need to repair oro-facial clefts early for cosmetic reasons, to preserve phonation and prevent other complications such as aspiration pneumonitis. The children referred from ENT were the oldest, probably since most were for adenotonsillectomy. It takes a while for adenoidal hypertrophy to reach levels that can cause obstructive sleep apnoea syndrome and therefore the need for surgical intervention.

The left ventricular function of the study population was adequate, irrespective of whether they had cardiac anomalies or not. This was demonstrated by the normal median values of the EF and FS in the study population and the lack of a significant difference between the median FS and EF of the children with cardiac anomalies and those without anomalies.

There are limitations to the interpretation of our results.It was a retrospective review with the attendant problems of missing records, poor documentation or insufficient clinical information. However the problems of missing records or poor documentation were obviated by the single source of echocardiography and uniform documentation of findings. Secondly, the observed prevalence of cardiac anomalies does not represent the prevalence in the community. Most of the children were delivered in the hospital or sought further care in our centre. Echo detection of cardiac anomalies in the children referred for pre-anaesthetic evaluation remains the strength of this study.

## Conclusion

Of the children referred for pre-anaesthetic echo evaluation,21.5% had cardiac anomalies. The 16.7% prevalence of CHD among children with oro-facial clefts was high. Children with cleft palate had a higher prevalence of CHD compared with those with cleft lip only. Suspected CRS was the commonest reason for ophthalmological referral and accounted for 66.7% of cases with CHD. There was a low prevalence of ventricular hypertrophy in children with AH, some of whom had CHD, prompting the need for continued pre-anaesthetic echo evaluations. The cardiac anomalies were mostly acyanotic CHD. The children with congenital anomalies from other surgical units were more likely to have a positive echocardiographic screening. It is therefore recommended that pre-anaesthetic echocardiographic evaluation should be continued for children, especially those with suspected CRS and oro-facial clefts, and those with congenital anomalies.
